# Apolipoprotein A‐IV concentrations and clinical outcomes in a large chronic kidney disease cohort: Results from the GCKD study

**DOI:** 10.1111/joim.13437

**Published:** 2022-01-04

**Authors:** Johannes P. Schwaiger, Barbara Kollerits, Inga Steinbrenner, Hansi Weissensteiner, Sebastian Schönherr, Lukas Forer, Fruzsina Kotsis, Claudia Lamina, Markus P. Schneider, Ulla T. Schultheiss, Christoph Wanner, Anna Köttgen, Kai‐Uwe Eckardt, Florian Kronenberg, for the GCKD Investigators

**Affiliations:** ^1^ Institute of Genetic Epidemiology Medical University of Innsbruck Innsbruck Austria; ^2^ Department of Internal Medicine Landeskrankenhaus Hall i.T. Hall in Tirol Austria; ^3^ Institute of Genetic Epidemiology Faculty of Medicine and Medical Center University of Freiburg Freiburg Germany; ^4^ Department of Medicine IV – Nephrology and Primary Care Faculty of Medicine and Medical Center University of Freiburg Freiburg Germany; ^5^ Department of Nephrology and Hypertension University Hospital Erlangen Friedrich‐Alexander‐Universität Erlangen‐Nürnberg Erlangen Germany; ^6^ Division of Nephrology Department of Internal Medicine I University Hospital Würzburg Würzburg Germany; ^7^ Department of Nephrology and Medical Intensive Care Charité – Universitätsmedizin Berlin Berlin Germany

**Keywords:** all‐cause mortality, apolipoprotein A‐IV, cardiovascular disease, heart failure, high risk population, prospective study

## Abstract

**Background:**

Chronic kidney disease (CKD) represents a chronic proinflammatory state and is associated with very high cardiovascular risk. Apolipoprotein A‐IV (apoA‐IV) has antiatherogenic, antioxidative, anti‐inflammatory and antithrombotic properties and levels increase significantly during the course of CKD.

**Objectives:**

We aimed to investigate the association between apoA‐IV and all‐cause mortality and cardiovascular outcomes in the German Chronic Kidney Disease study.

**Methods:**

This was a prospective cohort study including 5141 Caucasian patients with available apoA‐IV measurements and CKD. The majority of the patients had an estimated glomerular filtration rate (eGFR) of 30–60 ml/min/1.73m^2^ or an eGFR >60 ml/min/1.73m^2^ in the presence of overt proteinuria. Median follow‐up was 6.5 years. The association of apoA‐IV with comorbidities at baseline and endpoints during follow‐up was modelled adjusting for major confounders.

**Results:**

Mean apoA‐IV concentrations of the entire cohort were 28.9 ± 9.8 mg/dl. Patients in the highest apoA‐IV quartile had the lowest high‐sensitivity C‐reactive protein values despite the highest prevalence of diabetes, albuminuria and the lowest eGFR. Each 10 mg/dl higher apoA‐IV translated into lower odds of prevalent cardiovascular disease (1289 cases, odds ratio = 0.80, 95% confidence interval [CI] 0.72–0.86, *p* = 0.0000003). During follow‐up, each 10 mg/dl higher apoA‐IV was significantly associated with a lower risk for all‐cause mortality (600 cases, hazard ratio [HR] = 0.81, 95% CI 0.73–0.89, *p* = 0.00004), incident major adverse cardiovascular events (506 cases, HR = 0.88, 95% CI 0.79–0.99, *p* = 0.03) and death or hospitalizations due to heart failure (346 cases, HR = 0.84, 95% CI 0.73–0.96, *p* = 0.01).

**Conclusions:**

These data support a link between elevated apoA‐IV concentrations and reduced inflammation in moderate CKD. ApoA‐IV appears to be an independent risk marker for reduced all‐cause mortality, cardiovascular events and heart failure in a large cohort of patients with CKD.

## Graphical abstract

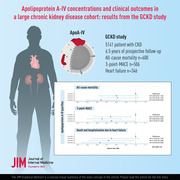

## Introduction

Chronic kidney disease (CKD) is a major public health problem affecting more than 10% of the general population in many countries worldwide [1]. This equals roughly 850 million individuals, a number that is twice as high as the number of people with diabetes [2]. CKD is considered a systemic inflammatory disease with many causes [3]. This proinflammatory state contributes to vascular and myocardial remodelling and accelerated aging resulting in atherosclerotic lesions and vascular and myocardial fibrosis. Therefore, CKD is—amongst other factors—associated with an exceptionally high cardiovascular disease (CVD) morbidity and mortality including a high risk for heart failure [4, 5, 6]. Recent research suggests that limiting inflammation in particular in patients with moderate CKD might be beneficial [7]. The leading biomarker for inflammation is C‐reactive protein (CRP). However, CRP is a signal for inflammation but has no causal role in atherosclerosis development [8].

Apolipoprotein A‐IV (apoA‐IV) is a 46 kDa glycoprotein involved in reverse cholesterol transport modulating lipid metabolism and glucose homeostasis [9, 10, 11]. It is produced in enterocytes and serves as a constituent of chylomicrons, very‐low‐density lipoprotein and high‐density lipoprotein (HDL), or remains not associated with lipoproteins [12, 13, 14]. ApoA‐IV has antiatherogenic, antioxidative, antithrombotic and anti‐inflammatory properties [15, 16, 17, 18, 19, 20, 21, 22, 23]. The experimental evidence for the anti‐inflammatory properties is quite strong: in a mouse model for acute colitis, apoA‐IV significantly and specifically delayed the onset and reduced the severity and extent of inflammation induced by dextran sulfate sodium. ApoA‐IV knockout mice exhibited a significantly greater inflammatory response to dextran sulfate sodium, which was reversed by exogeneous administration of apoA‐IV [23]. Furthermore, apolipoprotein E–deficient mice that overexpress human apoA‐IV showed significantly less atherosclerotic lesions. Lymphocytes of these animals treated with lipopolysaccharides to mimic chronic infection produced less proinflammatory cytokines and recombinant apoA‐IV blocked the lipopolysaccharide‐induced stimulation of monocytes [18].

Mice transgenic for human apoA‐IV are protected against atherosclerosis by a mechanism that does not involve an increase in HDL cholesterol concentration [16]. Recombinant apoA‐IV treatment was reported to be associated with a more stable plaque phenotype [17]. Data on apoA‐IV and atherosclerosis in humans are sparse, but suggest an inverse association of apoA‐IV concentrations with CVD [24, 25, 26, 27, 28]. Recent studies described a relationship between apoA‐IV glycation and coronary artery disease severity in patients with type‐2 diabetes mellitus [29].

Kidney function has a major influence on apoA‐IV concentrations. Concentrations increase with decreasing estimated glomerular filtration rate (eGFR) [26, 27], and apoA‐IV was shown to be an early marker of kidney impairment [30]. Furthermore, higher concentrations predicted the progression of CKD [31]. ApoA‐IV concentrations in dialysis patients have been reported to be twice as high compared to controls without CKD [32, 33]. In a study including more than 1200 haemodialysis patients with type‐2 diabetes mellitus, we reported that low apoA‐IV concentrations were strongly associated with a higher risk for all‐cause mortality, congestive heart failure and sudden cardiac death [34]. Prospective data on patients with CKD with moderately reduced eGFR and/or albuminuria, the overwhelming majority of patients with CKD, are lacking. We therefore aimed to examine the association between apoA‐IV and all‐cause mortality, incident CVD and heart failure in a large, prospective cohort study of 5141 patients with CKD, the German Chronic Kidney Disease (GCKD) study [35]. These patients can be considered as high‐risk patients for CVD.

## Methods

### GCKD study

The GCKD study is a prospective cohort study with ongoing follow‐up investigations including 5217 Caucasian patients with CKD enrolled between the years 2010 and 2012. The design and details of the study have been published previously [35, 36]. Briefly, the study aimed to enrol patients with an eGFR of 30–60 ml/min/1.73m^2^ (Kidney Disease Improving Global Outcomes [KDIGO] stage G3, A1–3) or an eGFR >60 ml/min/1.73m^2^ in the presence of overt proteinuria (KDIGO stage G1–2, A3) under regular care by nephrologists. Exclusion criteria were non‐Caucasian ethnicity, solid organ or bone marrow transplantation, active malignancy within 24 months prior to screening, heart failure New York Heart Association Stage IV, legal attendance or inability to provide consent. Patients were enrolled according to a standardised procedure by trained personnel. Blood and spot‐urine samples were collected, processed and shipped on dry ice to a central biobank where routine laboratory parameters were measured centrally, as reported previously [35]. eGFR was estimated using the CKD‐EPI formula.

The study was approved by the ethics committees of all participating institutions and registered in the national registry for clinical studies (DRKS 00003971). All methods were carried out in accordance with approved guidelines and the Declaration of Helsinki. Written informed consent was obtained from each study participant. Data are collected and managed using Askimed (https://www.askimed.com) as a cloud‐based web platform.

Patients are followed on a yearly basis by trained personnel who collect data on hospitalisations, outcome events and medical history using a structured interview. Any hospital discharge reports are collected from the treating physicians and/or hospitals. Endpoints are continually extracted from these reports by an endpoint committee. The prospectively collected endpoints considered in the present analysis refer to the first 6.5 years of follow‐up, based on data export from 8 October 2020.

### Definition of outcomes

CVD at baseline comprised a history of nonfatal myocardial infarction, coronary artery bypass grafting, percutaneous transluminal coronary angioplasty, stroke and interventions at the carotid arteries (carotid endarterectomy and/or carotid balloon angioplasty or stent implantation).

The prospectively collected endpoints considered in the present analysis refer to the first 6.5 years of follow‐up. All‐cause mortality was defined as the primary endpoint. Major adverse cardiovascular events (MACE), hospitalisations and death due to heart failure and further specific causes of death were considered secondary endpoints. MACE were either defined as 3‐point MACE including death from cardiovascular causes including myocardial infarction, coronary heart disease, decompensated congestive heart failure, sudden cardiac death and ischemic stroke as well as acute nonfatal myocardial infarction (ST‐elevation myocardial infarction, STEMI and non‐ST‐elevation myocardial infarction, NSTEMI), nonfatal stroke or as 4‐point MACE additionally including fatal peripheral ischemia, amputation due to peripheral vascular disease and surgical or percutaneous revascularisation due to peripheral vascular disease. Hospitalisation due to heart failure was defined as follows: hospitalisation with either evidence of a reduced left ventricular ejection fraction (<35%) or radiological evidence of pulmonary venous congestion, alveolar oedema or presence of bilateral or right‐sided pleural effusion with a presumed cardiac cause.

### Measurement of apoA‐IV plasma concentrations

Plasma apoA‐IV concentrations were measured at the Medical University of Innsbruck with a double‐antibody ELISA using an affinity‐purified polyclonal rabbit antihuman apoA‐IV antibody for coating. This antibody coupled with horseradish peroxidase was used for detection. Plasma with a known content of apoA‐IV served as the calibration standard. Patient samples were diluted 1:12500. In case of the rare situation of very low or very high concentrations outside of the linear range, samples were repeated using a lower or higher dilution, respectively. The intra‐ and inter‐assay coefficients of variation were 2.7% and 6%, respectively [31, 33, 34, 37].

### Statistical analysis

Baseline characteristics of the GCKD study participants are provided by quartiles of apoA‐IV concentrations. Jonckheere–Terpstra tests for trend were performed for comparison of continuous variables between quartile groups of apoA‐IV, and linear by linear χ^2^‐square tests were applied for comparison of categorical variables. Linear regression analysis was used to identify variables associated with apoA‐IV concentrations. Association with prevalent CVD was assessed with logistic regression analysis. Cause‐specific hazard ratios (HRs) and corresponding 95% confidence intervals (CIs) were calculated for all‐cause mortality, noncardiovascular mortality, incident cardiovascular endpoints and death or hospitalisation due to heart failure using Cox proportional hazards regression models. Within this approach, for the cause‐specific endpoints, all deaths from other causes were treated as censored observations. The proportional hazards assumption was tested by χ^2^‐test based on Schoenfeld residuals. In addition, for noncardiovascular mortality, incident cardiovascular endpoints and death and hospitalisation due to heart failure subdistribution hazard ratios (SHRs) based on competing risks survival regression were calculated treating all other causes of death as competing events. For both baseline and follow‐up analyses, model 1 was adjusted for age, sex, eGFR and urine albumin–creatinine ratio (UACR). In model 2 serum albumin, low‐density lipoprotein (LDL) cholesterol, smoking status, diabetes mellitus, statin use, triglycerides, body mass index (BMI), systolic and diastolic blood pressure (and CVD at baseline in the Cox regression analyses) were considered as additional covariables. This selection was based on clinical reasons and by taking into account differences of variables between quartiles of apoA‐IV, as shown in Table 1, and associations of variables with apoA‐IV, as displayed in Table 2. Forest plots of HR for quartiles of apoA‐IV for all‐cause mortality, 3‐point MACE, 4‐point MACE and death and hospitalisation due to heart failure were generated based on model 2. Based on the continuous prospective net reclassification index (NRI) for a median follow‐up time of 6.5 years, it was evaluated if apoA‐IV concentrations contributed to a better risk classification of individuals in terms of major endpoints as compared to a model already including renal and cardiac risk factors or parameters. The NRI was considered significant if the 95% CIs, determined empirically across 100 subsampling runs, excluded zero. The advantage of the continuous NRI is that it does not depend on the arbitrary selection of specific risk categories, and any change in predicted risk in the correct direction is appropriate [38]. Analyses on NRI were done with the function *nricens* in R. Due to the skewed distribution UACR, high‐sensitivity C‐reactive protein (hs‐CRP) and triglycerides were log transformed based on the natural logarithm (ln). Estimates were given for an increment of 10 mg/dl in apoA‐IV concentrations, as well as for quartile groups of apoA‐IV (using quartile 1 as reference). Further, a formal mediation analysis applying the product method approach [39] was performed, assuming hs‐CRP as a mediating variable. These models were adjusted as in model 2 and 95% CIs were derived via bootstrap sampling using 1000 iterations. Finally, a time‐dependent Cox proportional hazards model was applied as a sensitivity analysis. All variables where several measurements for a patient during the complete observation time were available were taken further for analysis (for more details, see [40]).

**Table 1:** Baseline characteristics of German Chronic Kidney Disease study patients stratified by quartiles of apolipoprotein A‐IV

	Apolipoprotein A‐IV quartiles				
	Quartile 1 (n = 1285)	Quartile 2 (n = 1286)	Quartile 3 (n = 1285)	Quartile 4 (n = 1285)	*p*‐value for trend
ApoA‐IV (mg/dl): range Mean ± SD 25th, 50th and 75th percentile	5.2–22.0 18.1 ± 3.0 (16.2; 18.6; 20.5)	22.0–27.6 24.8 ± 1.6 (23.4; 24.8; 26.1)	27.6–34.0 30.6 ± 1.8 (29.0; 30.4; 32.1)	34.0–100.2 42.0 ± 7.6 (36.5; 39.8; 45.1)	‐
Age (years)	60 ± 12 (52; 63; 70)	61 ± 12 (55; 65; 70)	61 ± 12 (55; 64; 70)	59 ± 12 (51; 62; 69)	0.005
Female gender, n (%)	588 (46)	501 (39)	484 (38)	477 (37)	<0.001
Body mass index (kg/m^2^)	30.3 ± 6.1 (26.2; 29.7; 33.7)	30.1 ± 6.0 (25.9; 29.1; 33.5)	29.8 ± 6.0 (25.9; 28.8; 33;1)	29.0 ± 5.6 (25.0; 28.2; 32.4)	<0.001
Smoker and ex‐smoker, n (%)	716 (56)	726 (56)	773 (60)	808 (63)	<0.001
Diabetes, n (%)	385 (30)	441 (34)	477 (37)	526 (41)	<0.001
Hypertension, n (%)	1195 (93)	1242 (97)	1242 (97)	1271 (99)	<0.001
Cardiovascular disease, n (%)	338 (26)	323 (25)	363 (28)	298 (23)	0.26
eGFR (ml/min/1.73 m^2^)	58 ± 20 (44; 54; 67)	51 ± 17 (40; 49; 59)	47 ± 16 (36; 44; 54)	42 ± 14 (31; 40; 48)	<0.001
Statin use, n (%)	549 (43)	568 (44)	631 (49)	693 (54)	<0.001
UACR (mg/g)	198 ± 585 (6; 19; 105)	274 ± 712 (7; 31; 193)	428 ± 973 (11; 55; 383)	814 ± 1284 (39; 281; 1075)	<0.001
Serum albumin (g/L)	39.0 ± 4.0 (36.8; 39.3; 41.2)	38.8 ± 4.0 (36.9; 39.1; 41.1)	38.3 ± 4.6 (36.3; 38.5; 40.6)	37.3 ± 4.8 (35.2; 37.8; 40.3)	<0.001
Haemoglobin (g/dl)	13.9 ± 1.7 (12.8; 13.8; 15.0)	13.8 ± 1.6 (12.8; 13.8; 14.9)	13.6 ± 1.6 (12.5; 13.5; 14.7)	13.2 ± 1.7 (12.1; 13.1; 14.3)	<0.001
Hs‐CRP (mg/L)	6.7 ± 12.8 (1.3; 2.9; 7.1)	4.7 ± 6.9 (1.1; 2.3; 5.1)	4.2 ± 6.2 (1.0; 2.2; 4.6)	3.4 ± 4.7 (0.8; 1.9; 4.0)	<0.001
Total cholesterol (mg/dl)	204 ± 47 (171; 203; 232)	210 ± 50 (176; 208; 240)	209 ± 50 (174; 206; 238)	221 ± 61 (181; 213; 251)	<0.001
LDL cholesterol (mg/dl)	116 ± 39 (88; 112; 141)	119 ± 42 (91; 115; 143)	116 ± 42 (89; 113; 141)	122 ± 50 (89; 115; 148)	0.14
HDL cholesterol (mg/dl)	50 ± 16 (38; 47; 58)	51 ± 16 (39; 47; 59)	51 ± 18 (39; 48; 61)	56 ± 21 (42; 52; 67)	<0.001
Triglycerides (mg/dl)	183 ± 104 (113; 160; 223)	199 ± 119 (122; 168; 239)	205 ± 138 (120; 175; 249)	209 ± 143 (117; 171; 257)	<0.001

*Note*: Values are provided as mean ± standard deviation and (25th; 50th [median]; and 75th percentiles) or as number of patients, n (%). In the total group, for all variables displayed, the number of missing values are ≤2.0% (n = 5141). eGFR (estimated glomerular filtration rate) calculated according to the CKD‐EPI equation. Hs‐CRP (high‐sensitivity C‐reactive protein) and urine‐albumin values that were below the lower detection limit (LOD) were replaced by LOD/√2. Body mass index was corrected for amputation. UACR (urine albumin–creatinine ratio) was calculated according to the following equation: albumin in urine (mg/l) × 100/creatinine in urine (mg/dl), and is given in mg/g. Hypertension was defined as systolic blood pressure ≥140 mmHg and/or diastolic blood pressure ≥90 mmHg, and/or receiving antihypertensive treatment. Cardiovascular disease was defined as myocardial infarction, coronary artery bypass grafting, percutaneous transluminal coronary angioplasty, stroke and/or interventions at the carotid arteries.

**Table 2:** Linear regression analysis investigating the influence of various clinical parameters on apolipoprotein A‐IV concentrations. All variables listed are included in the analysis at the same time

	β‐estimate	SE	*p*‐value
Age	−0.150	0.011	<0.001
Female sex	−1.705	0.261	<0.001
eGFR	−0.236	0.007	<0.001
Urine albumin–creatinine ratio^a^	0.002	0.0001	<0.001
Body mass index	−0.125	0.021	0.013
Current and ex‐smoker	0.733	0.241	0.002
Diabetes	3.019	0.263	<0.001
Statin use	0.783	0.234	0.001
HDL cholesterol	0.145	0.008	<0.001
Triglycerides^a^	0.007	0.001	<0.001
Hs‐CRP^a^	−0.147	0.014	<0.001

*Note*: Increment one unit for continuous variables. Triglycerides and hs‐CRP were ln‐transformed due to their skewed distribution.

Abbreviations: eGFR, estimated glomerular filtration rate; HDL, high‐density lipoprotein; Hs‐CRP, high‐sensitivity C‐reactive protein; SE, standard errors.

^a^ For better interpretability, beta estimates and SE were taken from the model without log transformation of skewed clinical parameters; *p*‐values are taken from the log‐transformed model.

Statistical analysis was performed using SPSS for Windows, version 26.0 (IBM Corp., Armonk, New York, NY, USA) and R for Windows, version 4.0.4 (Vienna, Austria) (https://www.r‐project.org). For all analyses performed, a two‐sided test *p*‐value <0.05 was considered statistically significant.

## Results

### Baseline analysis

**Fig 1:**
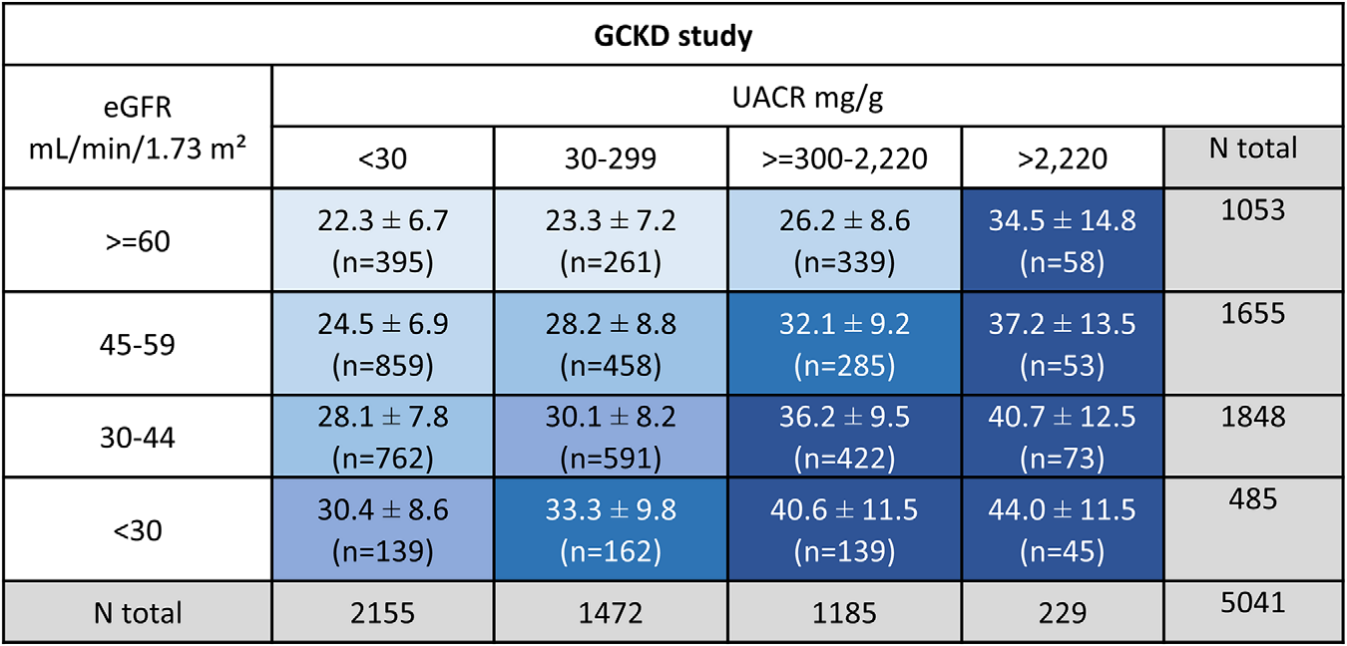
Mean (± standard deviation) apolipoprotein A‐IV (apoA‐IV) concentrations and number of patients stratified by estimated glomerular filtration rate (eGFR) and urine albumin–creatinine ratio (UACR) risk categories (including nephrotic range albuminuria >2220 mg/g) according to Kidney Disease Improving Global Outcomes guidelines in the German Chronic Kidney Disease (GCKD) study. Increasing concentrations of apoA‐IV are displayed with cell backgrounds in increasingly darker shades of blue (increment: 2.5 mg/dl in apoA‐IV concentration). Note: numbers of patients do not add up to the total number from GCKD with available apoA‐IV values due to missing values for eGFR and UACR.

Baseline apoA‐IV concentrations of patients were available in 5141 of 5217 patients included in the GCKD study with a mean plasma concentration of 28.9 ± 9.8 mg/dl. Clinical characteristics of patients at study baseline according to quartiles of apoA‐IV are presented in Table 1. The most striking difference was the decreasing eGFR (and increasing UACR) alongside increasing apoA‐IV levels. This became even more evident when apoA‐IV concentrations were assessed in the context of the KDIGO risk classification system based on cross categories of eGFR and UACR ('heat map', Fig. 1).

The median hs‐CRP concentrations in patients in the highest quartile of apoA‐IV concentrations were significantly lower as in the quartile with the lowest apoA‐IV concentrations (1.9 mg/L interquartile range [IQR] [0.8; 4.0] vs. 2.9 mg/L IQR [1.3; 7.1], *p* < 0.001) (Table 1). This was surprising considering that this quartile included patients who were already markedly more advanced in terms of kidney impairment and other clinical characteristics.

Variables independently associated with apoA‐IV concentrations are shown in Table 2. Besides age and sex, a lower eGFR, an increased UACR as well as increased HDL‐C and triglyceride concentrations were significantly associated with increasing apoA‐IV concentrations. The presence of diabetes mellitus was associated with 3 mg/dl higher apoA‐IV concentrations, whereas increased hs‐CRP concentrations and an elevated BMI were associated with decreasing apoA‐IV concentrations.

### CVD at baseline

We analysed the association between apoA‐IV concentrations and prevalent CVD. A total of 1289 of the 5141 patients had already experienced a CVD event before baseline and there was a strong inverse association with apoA‐IV concentrations: in the age‐, sex‐, eGFR‐ and UACR‐adjusted model, each increment of apoA‐IV by 10 mg/dl revealed 15% lower odds for prevalent CVD (odds ratio [OR] = 0.85, 95% CI 0.78–0.92, *p* = 0.0001). This association remained stable with an extended adjustment for serum albumin, LDL cholesterol, smoking status, diabetes mellitus, statin use, triglycerides, BMI and systolic and diastolic blood pressure: OR = 0.80, 95% CI 0.72–0.86, *p* = 0.0000003 (Table 3). This holds even true when hs‐CRP was added to the analysis (Table S1). When apoA‐IV was categorised in quartiles, the probability for prevalent CVD decreased for apoA‐IV values above the 25% percentile for all three models, with the most significant result when the fourth quartile was compared to the first (Table 3 and Table S1).

**Table 3:** Association of apolipoprotein A‐IV with prevalent cardiovascular disease (1289 out of 5141 patients)

		OR	95% CI	*p*‐value
Calculations per 10 mg/dl increment of apoA‐IV concentrations				
Model 1		0.85	0.78–0.92	0.0001
Model 2		0.80	0.72–0.86	0.0000003
Calculations per quartile of apoA‐IV concentrations				
Model 1	Quartile 1	1.00		
	Quartile 2	0.80	0.66–0.97	0.02
	Quartile 3	0.90	0.74–1.09	0.28
	Quartile 4	0.72	0.58–0.89	0.003
Model 2	Quartile 1	1.00		
	Quartile 2	0.80	0.65–0.99	0.04
	Quartile 3	0.87	0.70–1.07	0.18
	Quartile 4	0.63	0.50–0.79	0.00008

*Note*: Model 1: adjusted for age, sex, estimated glomerular filtration rate and ln‐urine albumin–creatinine ratio.

*Note*: Model 2: as model 1 plus serum albumin, low‐density lipoprotein cholesterol, smoking status, diabetes mellitus, statin use, ln‐triglycerides, body mass index and systolic and diastolic blood pressure.

*Note*: Prevalent cardiovascular disease was defined as myocardial infarction, coronary artery bypass grafting, percutaneous transluminal coronary angioplasty, stroke and/or interventions at the carotid arteries.

Abbreviations: CI, confidence interval; OR, odds ratio.

### All‐cause mortality and noncardiovascular mortality

**Fig 2:**
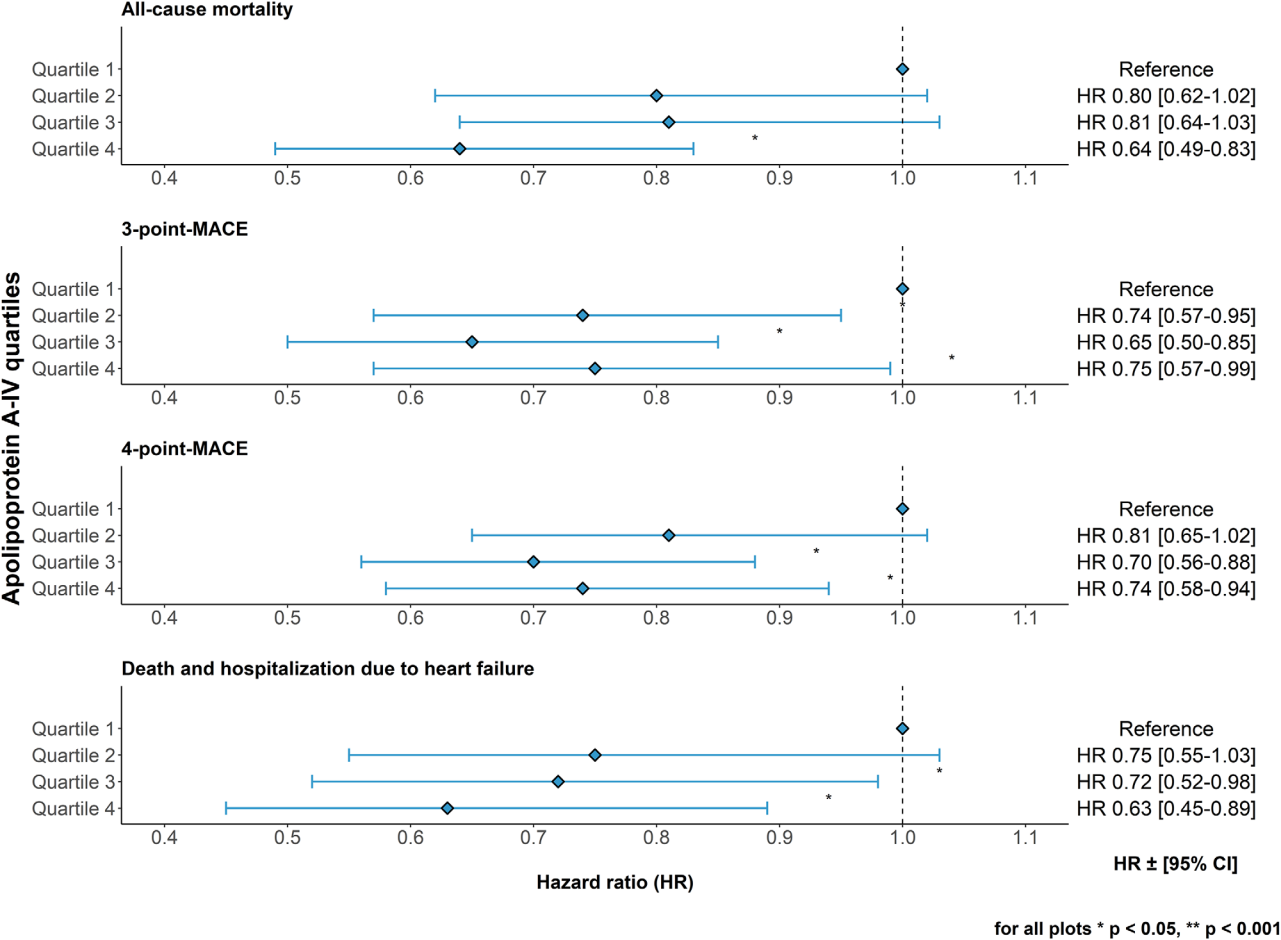
Forest plots for all‐cause mortality, 3‐point and 4‐point major adverse cardiovascular events (MACE) and death and hospitalisation due to heart failure by quartiles of apoA‐IV concentrations. Quartile 1 comprises patients with the lowest apoA‐IV concentrations. Data shown were adjusted for age, sex, estimated glomerular filtration rate, ln‐urine albumin–creatinine ratio, serum albumin, low‐density lipoprotein cholesterol, smoking status, diabetes mellitus, statin use, ln‐triglycerides, body mass index, systolic and diastolic blood pressure and cardiovascular disease at baseline. CI, confidence interval.

During a median follow‐up of 6.5 years, 600 out of 5041 patients (11.9%) with available apoA‐IV concentrations, eGFR, UACR and follow‐up data died. There was no indication for violation of the proportional hazards assumption in primary and secondary outcome analyses. Table 4 shows the results from Cox regression analyses. Each increase of apoA‐IV by 10 mg/dl was associated with a 17% lower risk to die during the observation period in the age‐, sex‐ and kidney function‐adjusted model 1 (HR = 0.83, 95% CI 0.75–0.92, *p* = 0.0003). This association remained significant in extended model 2 additionally adjusted for serum albumin, LDL cholesterol, smoking status, diabetes mellitus, statin use, triglycerides, BMI, systolic and diastolic blood pressure and CVD at baseline (HR = 0.81, 95% CI 0.73–0.89, *p* = 0.00004). An analysis according to quartiles of apoA‐IV mirrored the results from the continuous analysis, which, however, reached statistical significance only in quartile 4 and barely missed significance in quartiles 2 and 3 (Fig. 2, adjustment according to extended model 2). ApoA‐IV concentrations were also significantly associated with noncardiovascular mortality (Table 4).

**Table 4:** Association of apolipoprotein A‐IV with outcomes during the prospective follow‐up

	For each increase of apoA‐IV by 10 mg/dl	
Adjustment model	HR (95% CI)	*p*‐value
All‐cause mortality (n = 600)^a^		
Model 1	0.83 (0.75–0.92)	0.0003
Model 2	0.81 (0.73–0.89)	0.00004
Noncardiovascular mortality (n = 433)^a^		
Model 1	0.80 (0.71–0.90)	0.0003
Model 2	0.76 (0.68–0.86)	0.00001
3‐point MACE (n = 506)^a^		
Model 1	0.89 (0.80–0.99)	0.03
Model 2	0.88 (0.79–0.99)	0.03
4‐point MACE (n = 681)^a^		
Model 1	0.90 (0.82–0.99)	0.03
Model 2	0.88 (0.80–0.97)	0.01
Death and hospitalisation due to heart failure (n = 346)^a^		
Model 1	0.83 (0.72–0.95)	0.006
Model 2	0.84 (0.73–0.96)	0.01

*Note*: Model 1: adjusted for age, sex, estimated glomerular filtration rate and ln‐urine albumin–creatinine ratio.

*Note*: Model 2: as model 1 plus serum albumin, low‐density lipoprotein cholesterol, smoking status, diabetes mellitus, statin use, ln‐triglycerides, body mass index, systolic and diastolic blood pressure and cardiovascular disease at baseline.

*Note*: 3‐point MACE was defined as acute myocardial infarction (ST‐elevation myocardial infarction, STEMI and non‐ST‐elevation myocardial infarction, NSTEMI), nonfatal stroke, fatal myocardial infarction, fatal coronary heart disease, sudden cardiac death, death from congestive heart failure and death due to nonhaemorrhagic stroke.

*Note*: 4‐point MACE comprised all endpoints included in 3‐point MACE plus fatal peripheral ischemia, amputation due to peripheral vascular disease and surgical or percutaneous revascularisation due to peripheral vascular disease.

*Note*: Hospitalisation due to heart failure was defined as follows: hospitalisation with either evidence of a reduced left ventricular ejection fraction (< 35%) or radiological evidence of pulmonary venous congestion, alveolar oedema or presence of bilateral or right‐sided pleural effusion with a presumed cardiac cause.

Abbreviations: CI, confidence interval; HR, hazard ratio; MACE, major adverse cardiovascular events.

^a^ Number of events refers to model 1.

### MACE during the prospective follow‐up

We analysed all incident MACE using the 3‐point MACE (n = 506 patients with events) and 4‐point MACE (n = 681 patients) definitions (Fig. 2). Again, we observed 12% lower hazards with each 10 mg/dl higher apoA‐IV concentrations for both endpoints (Table 4, extended adjustment model 2). When data were analysed by quartiles, we observed decreasing risks with increasing apoA‐IV quartiles, which were significant in the second, third and fourth quartile for 3‐point MACE and for the third and fourth quartile for 4‐point MACE (Fig. 2, adjustment according to extended model 2).

### Death and hospitalisations due to heart failure during prospective follow‐up

A total of 346 patients were hospitalised at least once or died due to heart failure during the observation period. We observed strong associations of apoA‐IV levels with this outcome: each increase of apoA‐IV by 10 mg/dl was associated with a 16% lower risk in the extended adjustment model 2 (HR = 0.84, 95% CI 0.73–0.96, *p* = 0.01). When data were analysed by quartiles, we observed significantly lower risks in the third and fourth quartiles of apoA‐IV compared to the first quartile (Fig. 2, adjustment according to extended model 2).

### Mediation analyses

**Fig 3:**
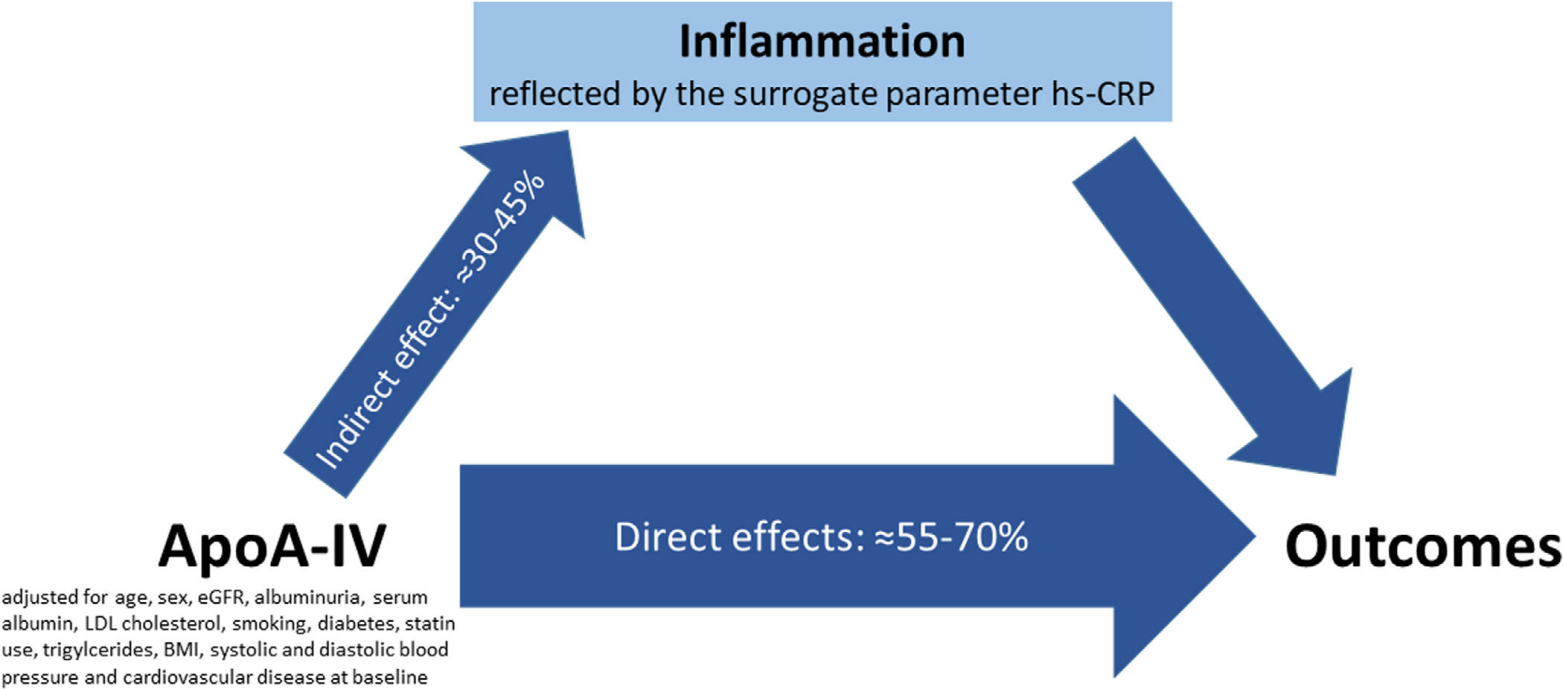
Results of mediation analysis, splitting the total effect of apoA‐IV on incident outcomes into indirect effects (proportion mediated by inflammation) and direct effects of apoA‐IV (i.e., proportion of apoA‐IV effects on outcomes, which is not mediated by inflammation). The thickness of the arrows is proportional to the proportion mediated.

Since experimental data strongly support the anti‐inflammatory properties of apoA‐IV, we added in a first step hs‐CRP as an indicator for inflammation to the extended model 2 of each outcome. The results remained significant for all‐cause mortality but were no longer significant for the incident cardiovascular endpoints and death and hospitalisation due to heart failure (Table S2). We therefore performed in a second step a formal mediation analysis to see whether and how much of the association of apoA‐IV with outcomes is mediated by inflammation and used hs‐CRP as a surrogate for the inflammatory state. For all outcomes, hs‐CRP significantly mediated the effect of apoA‐IV (already adjusted using the variables from adjustment model 2) by roughly 31% (all‐cause mortality) to 40%–44% (3‐point MACE, death and hospitalisation due to heart failure and 4‐point MACE in ascending order) (Fig. 3).

### Additional analyses

In a further analysis, we examined whether the association of apoA‐IV with prospective outcomes is influenced by the exclusion of the 1289 patients with prevalent CVD at the time of enrollment instead of adjusting for prevalent CVD. The HRs were only marginally influenced by these exclusions and remained significant for all‐cause mortality, 3‐point MACE and 4‐point MACE. The association was no longer significant for death and hospitalisation due to heart failure (Table S3).

For the prospective data analysis, we tested for an interaction with sex and found no indication for any interaction for all four endpoints (*p* for interaction >0.05). The estimates pointed in the same direction for both sexes.

The SHRs for 3‐point MACE and 4‐point MACE as well as death and hospitalisation due to heart failure were only slightly attenuated as compared to the cause‐specific HRs (Table S4).

In a sensitivity analysis, we additionally adjusted the analyses from Table 4 for HDL cholesterol, which only slightly weakened the association between apoA‐IV and outcomes (Table S5). This has been done due to the fact that a small fraction (12%–25%) of apoA‐IV is bound to HDL particles [12]. However, this adjustment has to be considered with caution since HDL cholesterol does not fulfil the classic requirements for a confounder since genetic studies have demonstrated the genetically explained HDL cholesterol concentrations are not associated with cardiovascular outcomes [41].

In a further sensitivity analysis, we added time‐updated covariates (eGFR, UACR, BMI, triglycerides, LDL cholesterol, serum albumin, systolic and diastolic blood pressure and statins) from follow‐up examinations 2, 3 and 4 years after baseline to the model. This resulted in virtually the same estimates for apoA‐IV concentrations (Table S6).

Finally, we analysed whether the addition of apoA‐IV to the analyses based on prospective continuous NRI showed a significant gain in classification accuracy. This was seen specifically for cases (between 9% and 11%) when apoA‐IV was added to the extended risk model 2 (including age, sex, eGFR, UACR, serum albumin, LDL cholesterol, smoking status, diabetes mellitus, statin use, triglycerides, BMI, systolic and diastolic blood pressure and CVD at baseline) concerning all four major endpoints: the NRI for cases for all‐cause mortality was 0.09 (95% CI: 0.04–0.16), for 3‐point MACE 0.07 (−0.001–0.17), 4‐point MACE 0.08 (0.02–0.15) and death and hospitalisations due to heart failure 0.11 (0.02–0.19).

## Discussion

### Main findings

This is the first large prospective study that has examined apoA‐IV concentrations in a cohort of high‐risk patients with moderate CKD. The main findings are: (i) patients in the highest apoA‐IV quartile had the lowest hs‐CRP values despite the lowest eGFR and the highest prevalence of diabetes and albuminuria, (ii) higher apoA‐IV concentrations at baseline were associated with lower odds of prevalent CVD, and (iii) during 6.5 years of follow‐up, higher apoA‐IV concentrations were associated with a lower risk of all‐cause mortality, MACE and heart failure. This was partly mediated by inflammation.

### Possible explanations

Oxidative stress, inflammation and atherosclerosis are closely linked [42, 43, 44] and are hallmarks not just only in patients with CKD who have an extraordinarily high risk to die especially from cardiovascular reasons. Most interestingly, we have observed that higher apoA‐IV levels were significantly associated with lower hs‐CRP levels, which could become the key for the understanding of this association: the quartile with the highest apoA‐IV levels showed roughly a third lower hs‐CRP concentrations than those in the quartile with the lowest apoA‐IV concentrations, although this quartile also comprised patients with a higher prevalence of hypertension, diabetes mellitus, smoking status, albuminuria and low eGFR (all together prominent causes of elevation of hs‐CRP [45]) and nevertheless having a lower risk for CVD events. In our analysis, we have also adjusted for statins—a drug group with proven anti‐inflammatory effect—with no relevant change in results. Malnutrition is another controversially discussed cause of inflammation and atherosclerosis, especially in dialysis patients [46, 47, 48]. However, our patients in the highest ApoA‐IV quartile had on average lower BMI and serum albumin values but nevertheless the lowest event rates and we adjusted for both variables in the regression models. Together, this is indeed surprising at the first glance. It suggests that the high apoA‐IV concentrations in this patient group might be a counteraction to the inflammatory burden and makes it unlikely that apoA‐IV is simply a negative acute phase reactant indicating good general health. The experimental evidence from various mouse models is very strong and in line with the idea that elevated apoA‐IV is acting anti‐inflammatory rather than being a consequence of inflammation, as mentioned in the introduction section [18, 23]. Furthermore, in the mediation analysis, we found that apoA‐IV already adjusted for traditional risk factors still has a strong association with the outcomes and roughly 30%–45% will be ‘transmitted’ by indirect effects on inflammation and 55%–70% by direct effects. Since hs‐CRP itself is not causally related to CVD outcomes, as shown by genetic studies [8], we used hs‐CRP as a surrogate for inflammation.

Besides or in combination with the antiatherogenic and antioxidative properties of apoA‐IV [16, 21], recombinant apoA‐IV stabilises the atherosclerosis plaque in the apoE‐knockout C57BL/6 mice and prevents acute plaque disruption [17]. Together with a further characteristic, apoA‐IV might contribute to the increased all‐cause mortality and MACE rate in patients with low apoA‐IV concentrations; Xu et al. [22] described that apoA‐IV negatively regulates αIIbβ3‐mediated platelet aggregation and therefore attenuates platelet aggregation and thrombosis. The authors found that the effect of apoA‐IV was similar to that of aspirin and clopidogrel, the two most commonly prescribed antiplatelet agents used in CVD prevention. Furthermore, thrombus formation was inhibited under both low‐ and high‐flow conditions simulating flow in both arterial and venous beds [22]. This antithrombotic effect might be an additional mechanism in how apoA‐IV could be protective against cardiovascular events. It needs to be seen whether apoA‐IV could be used for the treatment or prevention of atherosclerosis in humans.

Heart failure is a leading cardiovascular condition in patients with impaired kidney function [49, 50]. However, established left ventricular hypertrophy in patients with mild CKD is less common, but the prevalence is increasing with decreasing kidney function [51]. We have previously observed an association of low apoA‐IV concentrations with prevalent congestive heart failure in haemodialysis patients with type 2 diabetes [34]. In the study at hand, we not only extend these findings to patients with moderate CKD but can observe this association in a prospective study. The reason for this association remains unclear. Since inflammation is known to play a role in the left ventricular hypertrophy of CKD patients [52, 53], one may speculate that an anti‐inflammatory effect of apoA‐IV could counteract this effect of inflammation.

### Role of the kidney for the regulation of apoA‐IV concentrations

The average apoA‐IV concentrations in the general population are between 15 and 18 mg/dl [30]. We know from studies in the general population that apoA‐IV concentrations are an early marker of kidney impairment: concentrations start to increase early when eGFR decreases and albuminuria increases [30] and reach concentrations between 30 and 50 mg/dl in patients with kidney failure [33, 34]. Immunohistochemical studies suggested that apoA‐IV is filtered by the glomerulus and is reabsorbed by tubular cells. Therefore, serum levels increase when glomerular filtration declines [54], which is one explanation for the high apoA‐IV concentrations in CKD patients. Another explanation might be the above discussed response to the increasing oxidative and inflammatory burden of kidney disease [42, 55], indicating that apoA‐IV might be a risk modifier.

### Strengths and limitations of the study

Strengths of the study include the large sample size based on a well‐defined population with a median follow‐up of 6.5 years with almost no loss to follow‐up, homogeneity of the study population and a centralised assessment of the clinical outcomes. Limitations are: first, the observational design does not allow for clarifying causality or biological mechanisms. In case the association of apoA‐IV with outcomes is not causal—which cannot be proven or disproven in epidemiological studies—apoA‐IV is at least an adequate predictor of risk for these outcomes. However, the experimental data from many earlier studies are in strong support of a causal involvement [16, 18, 21–23]. Second, although our analyses were extensively adjusted for traditional cardiovascular risk factors, parameters of kidney function and statin use, we cannot exclude residual confounding by unknown or unmeasured variables, which might possibly show a causal relationship with both apoA‐IV and the outcomes. Some of those unmeasured variables or confounders are other surrogate measurements for inflammation such as interleukin‐6 which, unfortunately, are not available in the GCKD study. Third, it is unclear whether our findings are also valid for non‐Caucasian ethnicities. Fourth, the study mainly recruited CKD patients in stage G3 or A3 and the findings might not be generalisable to other stages of CKD or to general non‐CKD populations. However, our earlier study in diabetic haemodialysis patients revealed results pointing in a similar direction [34].

## Conclusions

This large prospective study in patients with moderate CKD revealed an independent and strong association of low apoA‐IV concentrations with all‐cause mortality and significant associations with prevalent and incident major cardiovascular events as well as heart failure. These associations might be driven by the known antioxidative, anti‐inflammatory and antithrombotic properties of this apolipoprotein. Further studies are required to elucidate the value of apoA‐IV as an anti‐inflammatory and antiatherogenic parameter in the general population and various clinical settings.

## Author contributions

Johannes P. Schwaiger: conceptualization; investigation; writing – original draft; writing – review and editing. Barbara Kollerits: conceptualization; data curation; formal analysis; methodology; visualization; writing – original draft; writing – review and editing. Inga Steinbrenner: data curation; investigation; validation; writing – review and editing. Hansi Weissensteiner: data curation; methodology; software; writing – review and editing. Sebastian Schönherr: data curation; investigation; software; writing – review and editing. Lukas Forer: data curation; investigation; software; writing – review and editing. Fruzsina Kotsis: data curation; investigation; validation; writing – review and editing. Claudia Lamina: formal analysis; methodology; validation; writing – review and editing. Markus P. Schneider: investigation; validation; writing – review and editing. Ulla T. Schultheiss: data curation; investigation; methodology; writing – review and editing. Christoph Wanner: conceptualization; data curation; funding acquisition; investigation; validation; writing – review and editing. Anna Köttgen: data curation; funding acquisition; investigation; supervision; writing – review and editing. Kai‐Uwe Eckardt: conceptualization; funding acquisition; investigation; resources; writing – review and editing. Florian Kronenberg: conceptualization; data curation; funding acquisition; investigation; project administration; resources; supervision; validation; writing – original draft; writing – review and editing.

## Supporting information




**Table S1** Association of apolipoprotein A‐IV with prevalent cardiovascular disease. Data are as in model 2 from Table 3 but additionally adjusted for hs‐CRP.
 **Table S2** Association of apolipoprotein A‐IV with outcomes during the prospective follow‐up. Data are as in model 2 from Table 4 but additionally adjusted for hs‐CRP.
 **Table S3** Association of apolipoprotein A‐IV with outcomes during the prospective follow‐up in patients free of cardiovascular disease at the time of enrollment (n = 3,852 out of 5,141). Data are adjusted for age, sex, eGFR, ln‐UACR, serum albumin, LDL cholesterol, smoking status, diabetes mellitus, statin use, ln‐triglycerides, BMI, systolic and diastolic blood pressure.
 **Table S4** Association of apolipoprotein A‐IV with outcomes during the prospective follow‐up based on subdistribution hazard ratio (SHR) models.
 **Table S5** Association of apolipoprotein A‐IV with outcomes during the prospective follow‐up when data are additionally adjusted for HDL cholesterol concentrations. For better comparison data from model 2 given in Table 4 of the main manuscript are added to this table.
**Table S6** Association of apolipoprotein A‐IV with outcomes during the prospective follow‐up including time‐updated covariables for eGFR, UACR, BMI, triglycerides, LDL cholesterol, serum albumin, systolic and diastolic blood pressure and statins from follow‐up examinations two, three and four years after baseline.

## Appendix

Current GCKD investigators and collaborators with the GCKD study are as follows: University of Erlangen‐Nürnberg: Kai‐Uwe Eckardt, Heike Meiselbach, Markus P. Schneider, Mario Schiffer, Hans‐Ulrich Prokosch, Barbara Bärthlein, Andreas Beck, André Reis, Arif B. Ekici, Susanne Becker, Dinah Becker‐Grosspitsch, Ulrike Alberth‐Schmidt, Birgit Hausknecht, Anke Weigel; University of Freiburg: Gerd Walz, Anna Köttgen, Ulla T. Schultheiß, Fruzsina Kotsis, Simone Meder, Erna Mitsch, Ursula Reinhard; RWTH Aachen University: Jürgen Floege, Turgay Saritas; Charité, University Medicine Berlin: Elke Schaeffner, Seema Baid‐Agrawal, Kerstin Theisen; Hannover Medical School: Hermann Haller, Jan Menne; University of Heidelberg: Martin Zeier, Claudia Sommerer, Johanna Theilinger; University of Jena: Gunter Wolf, Martin Busch, Rainer Paul; Ludwig‐Maximilians University of München: Thomas Sitter; University of Würzburg: Christoph Wanner, Vera Krane, Antje Börner‐Klein, Britta Bauer; Medical University of Innsbruck, Institute of Genetic Epidemiology: Florian Kronenberg, Julia Raschenberger, Barbara Kollerits, Lukas Forer, Sebastian Schönherr, Hansi Weissensteiner; University of Regensburg, Institute of Functional Genomics: Peter Oefner, Wolfram Gronwald; University of Bonn, Institute of Medical Biometry, Informatics and Epidemiology, Medical Faculty: Matthias Schmid, Jennifer Nadal.

### Conflict of interest

The authors declare no competing interests.

## References

[1] Eckardt K‐U , Coresh J , Devuyst O , Johnson RJ , Kottgen A , Levey AS , et al. Evolving importance of kidney disease: from subspecialty to global health burden. Lancet. 2013;382:158–69. 23727165 10.1016/S0140-6736(13)60439-0

[2] Jager KJ , Kovesdy C , Langham R , Rosenberg M , Jha V , Zoccali C . A single number for advocacy and communication‐worldwide more than 850 million individuals have kidney diseases. Nephrol Dial Transplant. 2019;34:1803–5. 31566230 10.1093/ndt/gfz174

[3] Jankowski J , Floege J , Fliser D , Bohm M , Marx N . Cardiovascular disease in chronic kidney disease: pathophysiological insights and therapeutic options. Circulation. 2021;143:1157–72. 33720773 10.1161/CIRCULATIONAHA.120.050686 PMC7969169

[4] Gansevoort RT , Correa‐Rotter R , Hemmelgarn BR , Jafar TH , Heerspink HJ , Mann JF , et al. Chronic kidney disease and cardiovascular risk: epidemiology, mechanisms, and prevention. Lancet. 2013;382:339–52. 23727170 10.1016/S0140-6736(13)60595-4

[5] Kronenberg F , Schernthaner GH . How many more data is required to give the kidney the attention it deserves? Time to act for the "big five" of cardiovascular risk. Atherosclerosis. 2020;297:146–8. 32057405 10.1016/j.atherosclerosis.2020.01.022

[6] Kottgen A , Russell SD , Loehr LR , Crainiceanu CM , Rosamond WD , Chang PP , et al. Reduced kidney function as a risk factor for incident heart failure: the atherosclerosis risk in communities (ARIC) study. J Am Soc Nephrol. 2007;18:1307–15. 17344421 10.1681/ASN.2006101159

[7] Ridker PM , Everett BM , Thuren T , MacFadyen JG , Chang WH , Ballantyne C , et al. Anti‐inflammatory therapy with canakinumab for atherosclerotic disease. N Engl J Med. 2017;377:1119–31. 28845751 10.1056/NEJMoa1707914

[8] Zacho J , Tybjaerg‐Hansen A , Jensen JS , Grande P , Sillesen H , Nordestgaard BG . Genetically elevated C‐reactive protein and ischemic vascular disease. N Engl J Med. 2008;359:1897–908. 18971492 10.1056/NEJMoa0707402

[9] Kohan AB , Wang F , Lo CM , Liu M , Tso P . ApoA‐IV: current and emerging roles in intestinal lipid metabolism, glucose homeostasis, and satiety. Am J Physiol Gastrointest Liver Physiol. 2015;308:G472–81. 25591862 10.1152/ajpgi.00098.2014 PMC4360046

[10] Kronenberg F . Apolipoprotein L1 and apolipoprotein A‐IV and their association with kidney function. Curr Opin Lipidol. 2017;28:39–45. 27870653 10.1097/MOL.0000000000000371

[11] Wang F , Kohan AB , Kindel TL , Corbin KL , Nunemaker CS , Obici S , et al. Apolipoprotein A‐IV improves glucose homeostasis by enhancing insulin secretion. Proc Natl Acad Sci U S A. 2012;109:9641–6. 22619326 10.1073/pnas.1201433109 PMC3386109

[12] Ezeh B , Haiman M , Alber HF , Kunz B , Paulweber B , Lingenhel A , et al. Plasma distribution of apoA‐IV in patients with coronary artery disease and healthy controls. JLR. 2003;44:1523–9. 10.1194/jlr.M300060-JLR200 12777472

[13] Green PHR , Glickman RM , Riley JW , Qinet E . Human apolipoprotein A‐IV. Intestinal origin and distribution in plasma. J Clin Invest. 1980;65:911–9. 6987270 10.1172/JCI109745 PMC434480

[14] Wang F , Kohan AB , Lo CM , Liu M , Howles P , Tso P . Apolipoprotein A‐IV: a protein intimately involved in metabolism. J Lipid Res. 2015;56:1403–18. 25640749 10.1194/jlr.R052753 PMC4513983

[15] Utermann G , Beisiegel U . Apolipoprotein A‐IV: a protein occurring in human mesenteric lymph chylomicrons and free in plasma. Isolation and quantification. Eur J Biochem. 1979;99:333–43. 115688 10.1111/j.1432-1033.1979.tb13261.x

[16] Duverger N , Tremp G , Caillaud JM , Emmanuel F , Castro G , Fruchart JC , et al. Protection against atherogenesis in mice mediated by human apolipoprotein A‐IV. Science. 1996;273:966–8. 8688083 10.1126/science.273.5277.966

[17] Geronimo FR , Barter PJ , Rye KA , Heather AK , Shearston KD , Rodgers KJ . Plaque stabilising effects of apolipoprotein A‐IV. Atherosclerosis. 2016;251:39–46. 27240254 10.1016/j.atherosclerosis.2016.04.019

[18] Recalde D , Ostos MA , Badell E , Garcia‐Otin AL , Pidoux J , Castro G , et al. Human apolipoprotein A‐IV reduces secretion of proinflammatory cytokines and atherosclerotic effects of a chronic infection mimicked by lipopolysaccharide. Arterioscler Thromb Vasc Biol. 2004;24:756–61. 14751811 10.1161/01.ATV.0000119353.03690.22

[19] Steinmetz A , Barbaras R , Ghalim N , Clavey V , Fruchart J‐C , Ailhaud G . Human apolipoprotein A‐IV binds to apolipoprotein A‐I/A‐ II receptor sites and promotes cholesterol efflux from adipose cells. J Biol Chem. 1990;265:7859–63. 2159462

[20] Stein O , Stein Y , Lefevre M , Roheim PS . The role of apolipoprotein A‐IV in reverse cholesterol transport studied with cultured cells and liposomes derived from another analog of phosphatidylcholine. Biochim Biophys Acta. 1986;878:7–13. 3089295 10.1016/0005-2760(86)90337-1

[21] Cohen RD , Castellani LW , Qiao JH , Van Lenten BJ , Lusis AJ , Reue K . Reduced aortic lesions and elevated high density lipoprotein levels in transgenic mice overexpressing mouse apolipoprotein A‐IV. J Clin Invest. 1997;99:1906–16. 9109435 10.1172/JCI119358 PMC508015

[22] Xu XR , Wang Y , Adili R , Ju L , Spring CM , Jin JW , et al. Apolipoprotein A‐IV binds alphaIIbbeta3 integrin and inhibits thrombosis. Nat Commun. 2018;9:3608. 30190457 10.1038/s41467-018-05806-0 PMC6127106

[23] Vowinkel T , Mori M , Krieglstein CF , Russell J , Saijo F , Bharwani S , et al. Apolipoprotein A‐IV inhibits experimental colitis. J Clin Invest. 2004;114:260–9. 15254593 10.1172/JCI21233 PMC450164

[24] Kronenberg F , Stühlinger M , Trenkwalder E , Geethanjali FS , Pachinger O , Von Eckardstein A , et al. Low apolipoprotein A‐IV plasma concentrations in men with coronary artery disease. J Am Coll Cardiol. 2000;36:751–7. 10987595 10.1016/s0735-1097(00)00775-0

[25] Mampuya MW , Guo J , Zhao Y . The relationship between plasma ap1olipoprotein A‐IV levels and coronary heart disease. Chin Med J (Engl). 2001;114:275–9. 11780313

[26] Kronenberg F , Kuen E , Ritz E , König P , Kraatz G , Lhotta K , et al. Apolipoprotein A‐IV serum concentrations are elevated in mild and moderate renal failure. J Am Soc Nephrol. 2002;13:461–9. 11805176 10.1681/ASN.V132461

[27] Lingenhel A , Lhotta K , Neyer U , Heid IM , Rantner B , Kronenberg MF , et al. Role of the kidney in the metabolism of apolipoprotein A‐IV: influence of the type of proteinuria. JLR. 2006;47:2071–9. 10.1194/jlr.M600178-JLR200 16788210

[28] Omori M , Watanabe M , Matsumoto K , Honda H , Hattori H , Akizawa T . Impact of serum apolipoprotein A‐IV as a marker of cardiovascular disease in maintenance hemodialysis patients. Ther Apher Dial. 2010;14:341–8. 20609189 10.1111/j.1744-9987.2010.00809.x

[29] Dai Y , Shen Y , Li QR , Ding FH , Wang XQ , Liu HJ , et al. Glycated apolipoprotein A‐IV induces atherogenesis in patients with CAD in type 2 diabetes. J Am Coll Cardiol. 2017;70:2006–19. 29025558 10.1016/j.jacc.2017.08.053

[30] Stangl S , Kollerits B , Lamina C , Meisinger C , Huth C , Stöckl A , et al. Association between apolipoprotein A‐IV concentrations and chronic kidney disease in two large population‐based cohorts: results from the KORA studies. J Intern Med. 2015;278:410–23. 26037138 10.1111/joim.12380

[31] Boes E , Fliser D , Ritz E , König P , Lhotta K , Mann JF , et al. Apolipoprotein A‐IV predicts progression of chronic kidney disease: the mild to moderate kidney disease study. J Am Soc Nephrol. 2006;17:528–36. 16382017 10.1681/ASN.2005070733

[32] Seishima M , Muto Y . An increased apo A‐IV serum concentration of patients with chronic renal failure on hemodialysis. Clin Chim Acta. 1987;167:303–11. 3621614 10.1016/0009-8981(87)90350-0

[33] Kronenberg F , König P , Neyer U , Auinger M , Pribasnig A , Lang U , et al. Multicenter study of lipoprotein(a) and apolipoprotein(a) phenotypes in patients with end‐stage renal disease treated by hemodialysis or continuous ambulatory peritoneal dialysis. J Am Soc Nephrol. 1995;6:110–20. 7579063 10.1681/ASN.V61110

[34] Kollerits B , Krane V , Drechsler C , Lamina C , März W , Ritz E , et al. Apolipoprotein A‐IV concentrations and clinical outcomes in haemodialysis patients with type 2 diabetes mellitus – a post hoc analysis of the 4D Study. J Intern Med. 2012;272:592–600. 22891946 10.1111/j.1365-2796.2012.02585.x

[35] Titze S , Schmid M , Kottgen A , Busch M , Floege J , Wanner C , et al. Disease burden and risk profile in referred patients with moderate chronic kidney disease: composition of the German Chronic Kidney Disease (GCKD) cohort. Nephrol Dial Transplant. 2015;30:441–51. 25271006 10.1093/ndt/gfu294

[36] Fazzini F , Lamina C , Raschenberger J , Schultheiss UT , Kotsis F , Schönherr S , et al. Results from the German Chronic Kidney Disease (GCKD) study support association of relative telomere length with mortality in a large cohort of patients with moderate chronic kidney disease. Kidney Int. 2020;98:488–97. 32641227 10.1016/j.kint.2020.02.034

[37] Kronenberg F , Lobentanz E‐M , König P , Utermann G , Dieplinger H . Effect of sample storage on the measurement of lipoprotein(a), apolipoproteins B and A‐IV, total and high‐density lipoprotein cholesterol and triglycerides. J Lipid Res. 1994;35:1318–28. 7964193

[38] Pencina MJ , D'Agostino RB, Sr. , Steyerberg EW . Extensions of net reclassification improvement calculations to measure usefulness of new biomarkers. Stat Med. 2011;30:11–21. 21204120 10.1002/sim.4085 PMC3341973

[39] Van Der weele T , Vansteelandt S . Mediation analysis with multiple mediators. Epidemiologic Methods. 2013;2:95–115. 10.1515/em-2012-0010 PMC4287269 25580377

[40] Zitt E , Lamina C , Sturm G , Knoll F , Lins F , Freistätter O , et al. Interaction of time‐varying albumin and phosphorus on mortality in incident dialysis patients. Clin J Am Soc Nephrol. 2011;6:2650–6. 21903986 10.2215/CJN.03780411 PMC3359564

[41] Kronenberg F . High‐density lipoprotein cholesterol on a roller coaster: where will the ride end? Kidney Int. 2016;89:747–9. 26994572 10.1016/j.kint.2016.01.007

[42] Cachofeiro V , Goicochea M , de Vinuesa SG , Oubina P , Lahera V , Luno J . Oxidative stress and inflammation, a link between chronic kidney disease and cardiovascular disease. Kidney Int Suppl. 2008;74:S4–S9. 10.1038/ki.2008.516 19034325

[43] Lawler PR , Bhatt DL , Godoy LC , Lüscher TF , Bonow RO , Verma S , et al. Targeting cardiovascular inflammation: next steps in clinical translation. Eur Heart J. 2021;42:113–31. 32176778 10.1093/eurheartj/ehaa099

[44] Hajjar DP , Gotto AM, Jr . Biological relevance of inflammation and oxidative stress in the pathogenesis of arterial diseases. Am J Pathol. 2013;182:1474–81. 23608224 10.1016/j.ajpath.2013.01.010 PMC3644714

[45] Ridker PM , MacFadyen J , Cressman M , Glynn RJ . Efficacy of rosuvastatin among men and women with moderate chronic kidney disease and elevated high‐sensitivity C‐reactive protein: a secondary analysis from the JUPITER (Justification for the Use of Statins in Prevention – an Intervention Trial Evaluating Rosuvastatin) trial. J Am Coll Cardiol. 2010;55:1266–73. 20206456 10.1016/j.jacc.2010.01.020

[46] Liu Y , Coresh J , Eustace JA , Longenecker JC , Jaar B , Fink NE , et al. Association between cholesterol level and mortality in dialysis patients: role of inflammation and malnutrition. JAMA. 2004;291:451–9. 14747502 10.1001/jama.291.4.451

[47] Stenvinkel P , Heimbürger O , Paultre F , Diczfalusy U , Wang T , Berglund L , et al. Strong association between malnutrition, inflammation, and atherosclerosis in chronic renal failure. Kidney Int. 1999;55:1899–911. 10231453 10.1046/j.1523-1755.1999.00422.x

[48] Ebert T , Qureshi AR , Lamina C , Fotheringham J , Froissart M , Eckardt KU , et al. Time‐dependent lipid profile inversely associates with mortality in hemodialysis patients – independent of inflammation/malnutrition. J Intern Med. 2021;290:910–21. 33998741 10.1111/joim.13291

[49] House AA , Wanner C , Sarnak MJ , Piña IL , McIntyre CW , Komenda P , et al. Heart failure in chronic kidney disease: conclusions from a Kidney Disease: Improving Global Outcomes (KDIGO) controversies conference. Kidney Int. 2019;95:1304–17. 31053387 10.1016/j.kint.2019.02.022

[50] Paoletti E , Specchia C , Di Maio G , Bellino D , Damasio B , Cassottana P , et al. The worsening of left ventricular hypertrophy is the strongest predictor of sudden cardiac death in haemodialysis patients: a 10 year survey. Nephrol Dial Transplant. 2004;19:1829–34. 15128888 10.1093/ndt/gfh288

[51] Paoletti E , De Nicola L , Gabbai FB , Chiodini P , Ravera M , Pieracci L , et al. Associations of left ventricular hypertrophy and geometry with adverse outcomes in patients with CKD and hypertension. Clin J Am Soc Nephrol. 2016;11:271–9. 26668021 10.2215/CJN.06980615 PMC4741044

[52] Yan L , Mathew L , Chellan B , Gardner B , Earley J , Puri TS , et al. S100/Calgranulin‐mediated inflammation accelerates left ventricular hypertrophy and aortic valve sclerosis in chronic kidney disease in a receptor for advanced glycation end products‐dependent manner. Arterioscler Thromb Vasc Biol. 2014;34:1399–411. 24855059 10.1161/ATVBAHA.114.303508 PMC4108264

[53] Gupta J , Dominic EA , Fink JC , Ojo AO , Barrows IR , Reilly MP , et al. Association between inflammation and cardiac geometry in chronic kidney disease: findings from the CRIC study. PLoS One. 2015;10:e0124772. 25909952 10.1371/journal.pone.0124772 PMC4409366

[54] Haiman M , Salvenmoser W , Scheiber K , Lingenhel A , Rudolph C , Schmitz G , et al. Immunohistochemical localisation of apolipoprotein A‐IV in human kidney tissue. Kidney Int. 2005;68:1130–6. 16105043 10.1111/j.1523-1755.2005.00519.x

[55] Shah SV , Baliga R , Rajapurkar M , Fonseca VA . Oxidants in chronic kidney disease. J Am Soc Nephrol. 2007;18:16–28. 17167116 10.1681/ASN.2006050500

