# Supplementary material for: Apolipoprotein A‐IV concentrations and clinical outcomes in a large chronic kidney disease cohort: Results from the GCKD study
Source: J Intern Med. 2022 Jan 4;291(5):622–36. doi: 10.1111/joim.13437 (PMC9305919; doi:10.1111/joim.13437)
Supplement: Supplementary file 1 — Table S1 Association of apolipoprotein A‐IV with prevalent cardiovascular disease. Data are as in model 2 from Table 3 but additionally adjusted for hs‐CRP. Table S2 Association of apolipoprotein A‐IV with outcomes during the prospective follow‐up. Data are as in model 2 from Table 4 but additionally adjusted for hs‐CRP. Table S3 Association of apolipoprotein A‐IV with outcomes during the prospective follow‐up in patients free of cardiovascular disease at the time of enrollment (n = 3,852 out of 5,141). Data are adjusted for age, sex, eGFR, ln‐UACR, serum albumin, LDL cholesterol, smoking status, diabetes mellitus, statin use, ln‐triglycerides, BMI, systolic and diastolic blood pressure. Table S4 Association of apolipoprotein A‐IV with outcomes during the prospective follow‐up based on subdistribution hazard ratio (SHR) models. Table S5 Association of apolipoprotein A‐IV with outcomes during the prospective follow‐up when data are additionally adjusted for HDL cholesterol concentrations. For better comparison data from model 2 given in Table 4 of the main manuscript are added to this table. Table S6 Association of apolipoprotein A‐IV with outcomes during the prospective follow‐up including time‐updated covariables for eGFR, UACR, BMI, triglycerides, LDL cholesterol, serum albumin, systolic and diastolic blood pressure and statins from follow‐up examinations two, three and four years after baseline. [file JOIM-291-622-s001.pdf]

Supplementary Tables to

Apolipoprotein A-IV concentrations and clinical outcomes in a large chronic kidney disease cohort: results from the GCKD study

Johannes P. Schwaiger [1,2], Barbara Kollerits [1], Inga Steinbrenner [3], Hansi Weissensteiner [1], Sebastian Schönherr [1], Lukas Forer [1], Fruzsina Kotsis [3,5], Claudia Lamina [1], Markus P. Schneider [4], Ulla T. Schultheiss [3,5], Christoph Wanner [6], Anna Köttgen [3], Kai-Uwe Eckardt [4,7], Florian Kronenberg [1]; for the GCKD Investigators

[1] Institute of Genetic Epidemiology, Medical University of Innsbruck, Innsbruck, Austria

[2] Department of Internal Medicine, Landeskrankenhaus Hall i.T., Hall i.T., Austria

[3] Institute of Genetic Epidemiology, Faculty of Medicine and Medical Center - University of Freiburg, Freiburg, Germany

[4] Department of Nephrology and Hypertension, University Hospital Erlangen, Friedrich-Alexander-Universität Erlangen-Nürnberg, Erlangen, Germany and German Chronic Kidney Disease study

[5] Department of Medicine IV – Nephrology and Primary Care, Faculty of Medicine and Medical Center - University of Freiburg, Freiburg, Germany

[6] Division of Nephrology, Department of Internal Medicine I, University Hospital Würzburg, Würzburg, Germany

[7] Department of Nephrology and Medical Intensive Care, Charité – Universitätsmedizin Berlin, Berlin, Germany

Corresponding author:

Florian Kronenberg, MD
Institute of Genetic Epidemiology
Department of Genetics and Pharmacology
Medical University of Innsbruck
Schöpfstrasse 41, A-6020 Innsbruck, Austria.
Phone: (+43) 512-9003-70560
Fax: (+43) 512 9003-73560 or -73561,
Email: Florian.Kronenberg@i-med.ac.at

Supplementary Table 1: Association of apolipoprotein A-IV with prevalent cardiovascular disease. Data are as in model 2 from Table 3 but additionally adjusted for hs-CRP.

	OR	95% CI	p-value
**Calculations per 10 mg/dL increment of apoA-IV concentrations**			
	0.83	(0.76-0.91)	$8x10^{-5}$
**Calculations per quartile of ApoA-IV concentrations**			
Quartile 1	1.00		
Quartile 2	0.84	(0.68-1.03)	0.10
Quartile 3	0.93	(0.76-1.15)	0.51
Quartile 4	0.71	(0.56-0.89)	0.004

OR, Odds ratio; CI, confidence interval

Data are adjusted for age, sex, eGFR, ln-UACR, serum albumin, LDL cholesterol, smoking status, diabetes mellitus, statin use, ln-triglycerides, BMI, systolic and diastolic blood pressure and ln-hs-CRP

Prevalent cardiovascular disease was defined as myocardial infarction, coronary artery bypass grafting, percutaneous transluminal coronary angioplasty, stroke, interventions at the carotid arteries.

Supplementary Table 2: Association of apolipoprotein A-IV with outcomes during the prospective follow-up. Data are as in model 2 from Table 4 but additionally adjusted for hs-CRP.

	For each increase of apoA-IV by 10 mg/dL	
**Endpoints**	**HR (95% CI)**	**p-value**
All-cause mortality	0.87 (0.78-0.97)	0.009
Non-cardiovascular mortality	0.82 (0.73-0.93)	0.002
3-point-MACE	0.93 (0.83-1.04)	0.21
4-point-MACE	0.94 (0.85-1.03)	0.18
Death and hospitalization due to heart failure	0.91 (0.79-1.04)	0.16

HR, hazard ratio; CI, confidence interval

Data are adjusted for age, sex, eGFR, ln-UACR, serum albumin, LDL cholesterol, smoking status, diabetes mellitus, statin use, ln-triglycerides, BMI, systolic and diastolic blood pressure, cardiovascular disease at baseline and ln-hs-CRP.

3-point-MACE (Major Adverse Cardiovascular Events) was defined as acute myocardial infarction (STEMI and NSTEMI), non-fatal stroke, fatal myocardial infarction, fatal coronary heart disease, sudden cardiac death, death from congestive heart failure and death due to non-hemorrhagic stroke;

4-point-MACE comprised all endpoints included in 3-point-MACE plus fatal peripheral ischemia, amputation due to peripheral vascular disease, surgical or percutaneous revascularization due to peripheral vascular disease

Hospitalization due to heart failure was defined as follows: hospitalization with either evidence of a reduced left ventricular ejection fraction (< 35%) or radiological evidence of pulmonary venous congestion, alveolar edema or presence of bilateral or right-sided pleural effusion with a presumed cardiac cause.

Supplementary Table 3: Association of apolipoprotein A-IV with outcomes during the prospective follow-up in patients free of cardiovascular disease at the time of enrollment (n=3,852 out of 5,141). Data are adjusted for age, sex, eGFR, ln-UACR, serum albumin, LDL cholesterol, smoking status, diabetes mellitus, statin use, ln-triglycerides, BMI, systolic and diastolic blood pressure.

	For each increase of apoA-IV by 10 mg/dL	
**Endpoints (n of events)**	**HR (95% CI)**	**p-value**
All-cause mortality (n=300)	0.84 (0.73-0.96)	0.009
Non-cardiovascular mortality (n=233)	0.78 (0.66-0.91)	0.002
3-point-MACE (n=235)	0.85 (0.73-0.99)	0.04
4-point-MACE (n=321)	0.88 (0.78-1.01)	0.06
Death and hospitalization due to heart failure (n=159)	0.87 (0.72-1.05)	0.15

HR, hazard ratio; CI, confidence interval

3-point-MACE (Major Adverse Cardiovascular Events) was defined as acute myocardial infarction (STEMI and NSTEMI), non-fatal stroke, fatal myocardial infarction, fatal coronary heart disease, sudden cardiac death, death from congestive heart failure and death due to non-hemorrhagic stroke;

4-point-MACE comprised all endpoints included in 3-point-MACE plus fatal peripheral ischemia, amputation due to peripheral vascular disease, surgical or percutaneous revascularization due to peripheral vascular disease

Hospitalization due to heart failure was defined as follows: hospitalization with either evidence of a reduced left ventricular ejection fraction (< 35%) or radiological evidence of pulmonary venous congestion, alveolar edema or presence of bilateral or right-sided pleural effusion with a presumed cardiac cause.

Supplementary Table 4: Association of apolipoprotein A-IV with outcomes during the prospective follow-up based on subdistribution hazard ratio (SHR) models

Adjustment model		For each increase of apoA-IV by 10 mg/dL SHR (95% CI)	p-value
**Non-cardiovascular mortality (n=433)***			
	Model 1	0.80 (0.71-0.91)	0.0009
	Model 2	0.77 (0.68-0.87)	0.00005
**3-point-MACE (n=506) ***			
	Model 1	0.91 (0.82-1.01)	0.09
	Model 2	0.91 (0.81-1.02)	0.097
**4-point-MACE (n=681) ***			
	Model 1	0.92 (0.84-1.01)	0.09
	Model 2	0.91 (0.83-1.00)	0.05
**Death and hospitalization due to heart failure (n=346) ***			
	Model 1	0.86 (0.74-0.99)	0.03
	Model 2	0.88 (0.76-1.01)	0.06

CI, confidence interval

* Number of events refer to model 1.

Model 1: adjusted for age, sex, eGFR, ln-UACR;

Model 2: as model 1 plus serum albumin, LDL cholesterol, smoking status, diabetes mellitus, statin use, ln-triglycerides, BMI, systolic and diastolic blood pressure and cardiovascular disease at baseline

3-point-MACE (Major Adverse Cardiovascular Events) was defined as acute myocardial infarction (STEMI and NSTEMI), non-fatal stroke, fatal myocardial infarction, fatal coronary heart disease, sudden cardiac death, death from congestive heart failure and death due to non-hemorrhagic stroke;

4-point-MACE comprised all endpoints included in 3-point-MACE plus fatal peripheral ischemia, amputation due to peripheral vascular disease, surgical or percutaneous revascularization due to peripheral vascular disease

Hospitalization due to heart failure was defined as follows: hospitalization with either evidence of a reduced left ventricular ejection fraction (< 35%) or radiological evidence of pulmonary venous congestion, alveolar edema or presence of bilateral or right-sided pleural effusion with a presumed cardiac cause.

Supplementary Table 5: Association of apolipoprotein A-IV with outcomes during the prospective follow-up when data are additionally adjusted for HDL cholesterol concentrations. For better comparison data from model 2 given in Table 4 of the main manuscript are added to this table.

	For each increase of apoA-IV by 10 mg/dL	
**Adjustment model**	**HR (95% CI)**	**p-value**
**All-cause mortality**		
Model 2 from Table 4	0.81 (0.73-0.89)	0.00005
Model 2 + HDL cholesterol	0.81 (0.73-0.90)	0.000098
**Non-cardiovascular mortality**		
Model 2 from Table 4	0.76 (0.68-0.86)	0.00001
Model 2 + HDL cholesterol	0.75 (0.66-0.85)	0.000005
**3-point-MACE**		
Model 2 from Table 4	0.88 (0.79-0.99)	0.03
Model 2 + HDL cholesterol	0.92 (0.82-1.03)	0.14
**4-point-MACE**		
Model 2 from Table 4	0.88 (0.80-0.97)	0.01
Model 2 + HDL cholesterol	0.91 (0.83-1.01)	0.07
**Death and hospitalization due to heart failure**		
Model 2 from Table 4	0.83 (0.72-0.95)	0.006
Model 2 + HDL cholesterol	0.85 (0.74-0.98)	0.02

HR, hazard ratio; CI, confidence interval

Model 2: taken from Table 4 and is adjusted for age, sex, estimated glomerular filtration rate , ln-urine albumin-creatinine ratio, serum albumin, LDL cholesterol, smoking status, diabetes mellitus, statin use, ln-triglycerides, body mass index, systolic and diastolic blood pressure and cardiovascular disease at baseline

Model 2 + HDL cholesterol: additional adjustment for HDL cholesterol

3-point-MACE (Major Adverse Cardiovascular Events) was defined as acute myocardial infarction (STEMI and NSTEMI), non-fatal stroke, fatal myocardial infarction, fatal coronary heart disease, sudden cardiac death, death from congestive heart failure and death due to non-hemorrhagic stroke;

4-point-MACE comprised all endpoints included in 3-point-MACE plus fatal peripheral ischemia, amputation due to peripheral vascular disease, surgical or percutaneous revascularization due to peripheral vascular disease

Hospitalization due to heart failure was defined as follows: hospitalization with either evidence of a reduced left ventricular ejection fraction (< 35%) or radiological evidence of pulmonary venous congestion, alveolar edema or presence of bilateral or right-sided pleural effusion with a presumed cardiac cause.

Supplementary Table 6: Association of apolipoprotein A-IV with outcomes during the prospective follow-up including time-updated covariables for eGFR, UACR, BMI, triglycerides, LDL cholesterol, serum albumin, systolic and diastolic blood pressure and statins from follow-up examinations two, three and four years after baseline.

	For each increase of apoA-IV by 10 mg/dL	
**Adjustment model**	**HR (95% CI)**	**p-value**
**All-cause mortality (n=606) ***		
Model 1	0.83 (0.75-0.91)	0.0006
Model 2	0.80 (0.72-0.88)	0.00001
**Non-cardiovascular mortality (n=438) ***		
Model 1	0.81 (0.72-0.92)	0.001
Model 2	0.77 (0.69-0.87)	0.00003
**3-point-MACE (n=510) ***		
Model 1	0.89 (0.80-0.99)	0.03
Model 2	0.86 (0.77-0.96)	0.008
**4-point-MACE (n=686) ***		
Model 1	0.89 (0.81-0.97)	0.01
Model 2	0.86 (0.78-0.94)	0.002
**Death and hospitalization due to heart failure (n=350) ***		
Model 1	0.76 (0.67-0.87)	0.0002
Model 2	0.75 (0.66-0.86)	0.00004

HR, hazard ratio; CI, confidence interval

* Number of events refer to model 1.

Model 1: adjusted for age, sex, estimated glomerular filtration rate (longitudinal), ln- urine albumin-creatinine ratio (longitudinal);

Model 2: as model 1 plus serum albumin (longitudinal), LDL cholesterol (longitudinal), smoking status, diabetes mellitus, statin use (longitudinal), ln-triglycerides (longitudinal), body mass index (longitudinal), systolic and diastolic blood pressure (longitudinal) and cardiovascular disease at baseline

3-point-MACE (Major Adverse Cardiovascular Events) was defined as acute myocardial infarction (STEMI and NSTEMI), non-fatal stroke, fatal myocardial infarction, fatal coronary heart disease, sudden cardiac death, death from congestive heart failure and death due to non-hemorrhagic stroke;

4-point-MACE comprised all endpoints included in 3-point-MACE plus fatal peripheral ischemia, amputation due to peripheral vascular disease, surgical or percutaneous revascularization due to peripheral vascular disease

Hospitalization due to heart failure was defined as follows: hospitalization with either evidence of a reduced left ventricular ejection fraction (< 35%) or radiological evidence of pulmonary venous congestion, alveolar edema or presence of bilateral or right-sided pleural effusion with a presumed cardiac cause.
